# Working hours and depressive symptoms: the role of job stress factors

**DOI:** 10.1186/s40557-018-0257-5

**Published:** 2018-07-13

**Authors:** Yeogyeong Yoon, Jia Ryu, Hyunjoo Kim, Chung won Kang, Kyunghee Jung-Choi

**Affiliations:** 1 Department of Occupational and Environmental Medicine, Ewha Womans University, School of Medicine, 1071, Anyangcheon-ro, Yangcheon-gu, Seoul, 07985 South Korea; 2 Department of Occupational and Environmental Medicine, Ewha Womans University Medical Center, 1071, Anyangcheon-ro, Yangcheon-gu, Seoul, 07985 South Korea

**Keywords:** Working hours, Depressive symptom, Job stress

## Abstract

**Background:**

South Korea is one of the countries with the longest working hours in the OECD countries. The aim of this study was to evaluate the effect of working hours on depressive symptoms and the role of job stress factors between the two variables among employees in South Korea.

**Methods:**

This study used data from the Korea Working Conditions Survey in 2014. Study subjects included 23,197 employees aged 19 years or older who work more than 35 h per week. Working hours were categorized into 35–39, 40, 41–52, 53–68, and more than 68 h per week. Depressive symptoms were assessed using the WHO’s Well-Being Index with a cut-off score of 13. We calculated prevalence ratios of depressive symptoms according to working hours using log-binomial regression. Through the percentage change in prevalence ratios, we identified the extent of the role of job stress factors that explain depressive symptoms.

**Results:**

The risks of depressive symptoms were significantly higher in people who worked 35–39 h per week (PR: 1.09, CI: 1.01–1.18), 53–68 h/week (PR: 1.21, CI: 1.16–1.25), and more than 68 h/week (PR: 1.14, CI: 1.07–1.21) than 40 h/week, after adjusting for confounding variables. Job stress explained the effects of long working hours on depressive symptoms in about 20–40% of the groups working more than 40 h/week. Among the factors of job stress, social support was 10–30%, which showed the highest explanatory power in all working hours. Reward explained 15–30% in the more than 52 h working group, and reward was the most important factor in the working group that exceeded 68 h.

**Conclusions:**

We showed the working hours could be an independent risk factor for depressive symptoms in employees. To improve workers’ mental health, it is important to strengthen social support in the workplace, to provide adequate rewards as they work, and ultimately to regulate the appropriate amount of working hours.

## Background

South Korea is one of the countries with the longest working hours in the Organization for Economic Cooperation and Development (OECD) countries. According to the OECD annual report, the OECD Employment Outlook 2017, the average number of working hours in South Korea in 2016 was 2069 h, the second highest rate after Mexico [1]. The average annual working hours in the OECD 35 countries is 1764 h, and Korean workers are working 305 more hours than the average number of worked by workers in all OECD countries.

There are studies on the effects of long hours of work on health, and most studies report that long hours of work negatively affect workers’ health [2]. Long working hours can be a risk factor of mental illness as well as cardiovascular disease [3, 4]. On the other hand, previous research suggests that job stress increases during long working hours [5], and job stress is associated with depressive symptoms [6, 7]. People with high job demands due to excessive workload or time pressure had twice the risk of major depression or generalized anxiety disorder compared to those with low job demands [6]. Also, Job stress due to high effort and low reward has had a negative impact on mental health [7]. However, there are few studies that explored the role of job stress factors between long working hours and depressive symptoms [8].

**Fig. 1:**
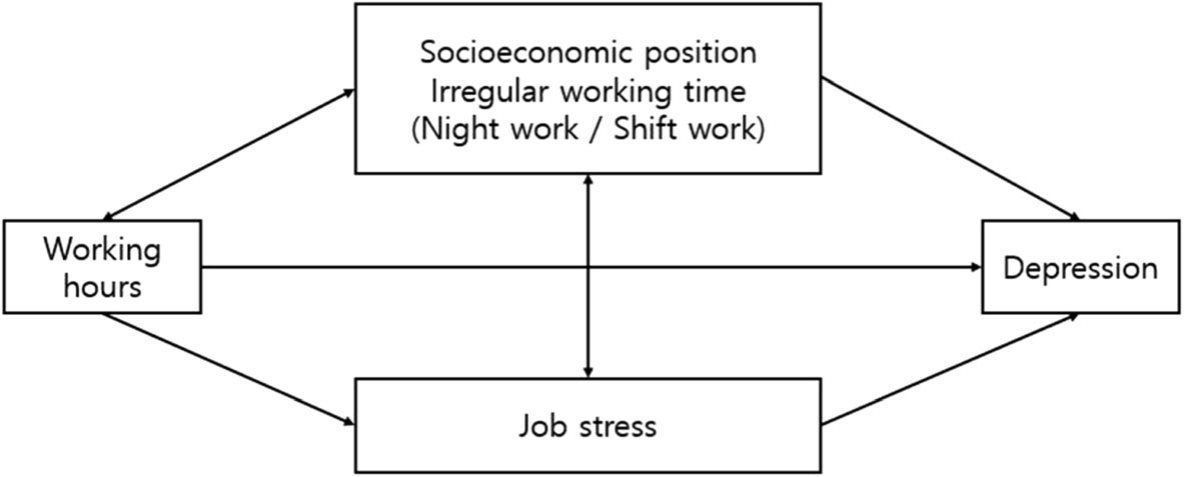
The outline of the relationship between working hours and depressive symptoms. Note. Socioeconomic position: education level, net monthly income, job category, employment status, Job stress: difficult physical environment, job autonomy, job demand, social support, and reward

The first purpose of this study is to evaluate the association of long working hours on depressive symptoms. The second purpose is to determine the magnitude of job stress factors explaining the pathways of long working hours affecting depressive symptoms, taking into account job stress as mediator. Figure 1 shows an outline of the relationship between working hours and depressive symptoms. Socioeconomic position and irregular working time were seen as confounders. Job stress is considered as a mediator because, as work hours increase, the hours for workers to be exposed to job stress become longer and stronger, and the characteristics of the job needing long or short working hours could be related to job stress.

## Materials and methods

### Data and study subjects

This study used data from the 4th Korea Working Conditions Survey (KWCS) in 2014. The survey was conducted by the Occupational Safety and Health Research Institute (OSHRI). The survey’s questionnaire was based on the 2010 European Working Conditions Survey (EWCS) and United Kingdom’s Labour Force Survey (LFS). Responses were collected from workers who were over 15 years old from a nationwide sample in Korea. The targeted sample size in the 4th KWCS was 50,000 and the number of total participants was 50,007. For the sample of the survey, a two-step probability proportion stratified cluster sample survey was used. The first survey extraction was based on the 2010 Population and Housing Census, and in the second extraction households and household members were selected. The response rate was 0.330, the cooperation ratio was 0.699, the rejection rate was 0.142 and the contact rate was 0.494 [9].

The study subjects were limited to employees excluding the self-employed, unpaid family workers, workers on childcare leave or other leave, and soldiers (19,353 people). The age of the subjects was limited to 19 years or older, so 120 persons were excluded. In addition, those who worked more than 35 h per week were selected and 7628 persons were excluded. Those who worked for less than 35 h are generally not likely to work five days a week. The lower limit of working hours was set to exclude selection bias, where health status affects job choice. Finally, the study included 23,197 employees over the age of 19 who worked over 35 h per week (Men 12,509 and women 10,688).

This study was approved by the Ewha Medical Center Institutional Review Board, Seoul, Korea (IRB FILE No. 2017–12-011).

### Variables

#### Working hours

The number of working hours were ascertained by a questionnaire that asked “How many hours do you usually work per week in your main paid job?” Working hours were categorized into 35–39, 40, 41–52, 53–68, and > 68 h per week. This categorization is based on the Korean Labor Standard Act. The Korean labor act sets 40 h per week and 8 h per day as legal working hours. Overtime work is possible up to 12 h per week with the consent of the employee. In addition, it is also possible to work 16 h (8 h each, two days) on weekends, apart from overtime work. The Ministry of Employment and Labor interpreted that it is possible to work up to 68 h per week because it does not recognize holiday work within 8 h as overtime work. Therefore, Korean employees can work up to 68 h. However, according to Article 59 of the Korean Labor Standards Act, which created a special provision for work and recess hours, it is possible to work overtime of 12 h per week for 26 industries such as transportation, communication, broadcasting and health. It also enables them to work over 68 h a week.

We define irregular working time as either shift work or night work. Shift work is categorized as yes or no. Night work means working at least two hours a day from 10:00 pm to 05:00 am more than once a month.

#### Depressive symptoms

Depressive symptoms were assessed using the 5-item WHO’s Well-Being Index (WHO-5) with a cut-off score of 13(total score 25). It is composed of 5 simple questions concerning emotions over the last two weeks. For instance, “I have felt cheerful and in good spirits.” Five questions were answered and scored to the Likert scaling from 0 to 5. A score below 13 indicates poor well-being and that is an indicator for a depression test under ICD-10. WHO-5 is a self-report questionnaire and a useful tool for screening for depression [10]. This scale is valid both for evaluating depression screening tools and for assessing subjective well-being [11].

#### Potential mediating variables and confounders

The work environment associated with job stress as a potential mediating variable includes variables of difficult physical environment, job autonomy, job demand, social support and reward. The tool for measuring socio-psychological work environment in KWCS was referred to Copenhagen Psychosocial Questionnaire (COPSOQ) II long version and Job Content Questionnaire (JCQ) at Eurofound (2012). The difficult physical environment was determined whether workers were exposed to physical, chemical, or ergonomic hazards by more than half of their working hours. Job autonomy investigated whether employees could choose or change one of the order, method, or speed of work. Job demand was rated 0–100 points as to whether it should work at a fast pace, whether it had a tight deadline, had enough time, and what was expected of workers. The higher the score, the higher the job demand. Social support classified as yes, no, and non-applicable for support from colleagues or managers. Reward was determined by how much workers agreed to whether they were well paid for the work.

Socioeconomic position and physical problems were selected as confounders. The socioeconomic position consists of education level (graduated high school or less, college, higher than graduated college), net monthly individual income (< 1 million, 1–1.99 million, 2–2.99 million or over 3 million KRW), job category and employment status (full-time employee, temporary employee or day employee). The employees consist of 10 occupations and their job categories were classified into three groups: white collar (administrator, professional, engineer and semi-professional, office worker), pink collar (service worker, sales worker), and blue collar (agriculture, forestry and fishery industry skilled worker, technical skilled worker and related skilled worker, equipment machinery operator and assembly worker, simple labor worker).

And we also checked on whether, over the last 12 months, the worker suffered from a physical problem (none or more than one). Physical problems include hearing problems, skin problems, backache, muscular pains, headaches, eyestrain, stomach ache, respiratory difficulties, cardiovascular diseases and injuries.

### Statistical analysis

This study used log-binomial regression to calculate the prevalence ratio in order to identify the association between working hours and depressive symptoms. We calculated the risk of depressive symptoms using the prevalence ratio (PR) instead of the odds ratio, because the incidence of depressive symptoms was as high as 30–60%.

The percentage change in PR was compared with the change in depressive symptoms according to the adjustment of potential mediating factors. Model 1 is the base model and is adjusted for age and gender. Model 2 is of the prevalence ratio adjusted for age, gender, education level, income, job category, employment status, physical problems and organization of working time. Model 2 and additional models were set up to compare the role of each mediating factor. Through the percentage change in PR, we examined whether our hypothesis has explanatory power (100 × [(PR in the baseline model (Model 2)) – (PR in the model adjusted for potential mediating variables)]/[(PR in the baseline model (Model 2))– 1]). All analyses were performed by using SAS statistical software (version 9.4, SAS institute, Cary, North Carolina, USA).

## Results

Table 1 shows the general characteristics of the study subjects. 45.3% of the total subjects worked 40 h per week. The proportions of workers who worked respectively 41–52, 53–68, > 68, and 35–39 h per week were 31.5, 15.7, 4.8, 2.8%. In men, the percentage of working over 68 h was more than twice that of women. The lower the level of education, the higher the rate of working over 53 h. In the occupation category, the proportion of pink and blue collar jobs over 53 h was higher than that of white collar jobs. Except for 40 h, the proportion of respondents who had physical problems was higher in the other working hours. 15.8% of the workers worked in irregular working time including shift work and night work. Among them, the rate of the irregular working group whose working time exceeds 68 h is 50.7%.

**Table 1:** General characteristics of study subjects N(%)

	Working hours					
	Total	35–39	40	41–52	53–68	> 68
Total	23,197 (100.0)	644 (2.8)	10,497 (45.3)	7299 (31.5)	3653 (15.7)	1104 (4.8)
Gender						
Men	12,509 (53.9)	182 (1.5)	5524 (44.2)	3931 (31.4)	2105 (16.8)	767 (6.1)
Women	10,688 (46.1)	462 (4.3)	4973 (46.5)	3368 (31.5)	1548 (14.5)	337 (3.2)
Age						
19–29	3025 (13.0)	109 (3.6)	1216 (40.2)	1029 (34.0)	569 (18.8)	102 (3.4)
30–39	6357 (27.4)	111 (1.7)	3139 (49.4)	2034 (32.0)	887 (14.0)	186 (2.9)
40–49	7062 (30.4)	160 (2.3)	3394 (48.1)	2219 (31.4)	1058 (15.0)	231 (3.3)
50–59	4743 (20.4)	141 (3.0)	2095 (44.2)	1445 (30.5)	820 (17.3)	242 (5.1)
60–69	1704 (7.3)	94 (5.5)	553 (32.5)	497 (29.2)	277 (16.3)	283 (16.6)
≥ 70	306 (1.3)	29 (9.5)	100 (32.7)	75 (24.5)	42 (13.7)	60 (19.6)
Education level						
Graduated high school or less	10,812 (46.6)	439 (4.1)	3701 (34.2)	3465 (32.0)	2349 (21.7)	858 (7.9)
College	11,833 (51.0)	190 (1.6)	6428 (54.3)	3716 (31.4)	1263 (10.7)	236 (2.0)
Higher than graduated college	552 (2.4)	15 (2.7)	368 (66.7)	118 (21.4)	41 (7.4)	10 (1.8)
Income (million KRW)						
< 1	1227 (5.3)	289 (23.6)	463 (37.7)	307 (25.0)	122 (9.9)	46 (3.7)
1–1.99	8940 (38.5)	210 (2.3)	3467 (38.8)	2943 (32.9)	1671 (18.7)	649 (7.3)
2–2.99	7091 (30.6)	82 (1.2)	3221 (45.4)	2363 (33.3)	1161 (16.4)	264 (3.7)
≥ 3	5939 (25.6)	63 (1.1)	3346 (56.3)	1686 (28.4)	699 (11.8)	145 (2.4)
Job category						
White collar	10,466 (45.1)	144 (1.4)	6438 (61.5)	3077 (29.4)	710 (6.8)	97 (0.9)
Pink collar	5735 (24.7)	269 (4.7)	1668 (29.1)	1894 (33.0)	1506 (26.3)	398 (6.9)
Blue collar	6996 (30.2)	231 (3.3)	2391 (34.2)	2328 (33.3)	1437 (20.5)	609 (8.7)
Employment status						
Full-time employee	18,860 (81.3)	305 (1.6)	9024 (47.8)	5984 (31.7)	2801 (14.9)	746 (4.0)
Temporary employee	3229 (13.9)	233 (7.2)	1107 (34.3)	995 (30.8)	609 (18.9)	285 (8.8)
Day employee	1108 (4.8)	106 (9.6)	366 (33.0)	320 (28.9)	243 (21.9)	73 (6.6)
Physical problem						
Yes	10,836 (46.7)	336 (3.1)	4314 (39.8)	3614 (33.4)	1961 (18.1)	611 (5.6)
No	12,361 (53.3)	308 (2.5)	6183 (50.0)	3685 (29.8)	1692 (13.7)	493 (4.0)
Irregular working time						
Yes	3672 (15.8)	98 (15.2)	1150 (11.0)	1152 (15.8)	712 (19.5)	560 (50.7)
No	19,525 (84.2)	546 (84.8)	9347 (89.0)	6147 (84.2)	2941 (80.5)	544 (49.3)

*Abbreviations:* Korean Won, KRW

Table 2 shows a distribution of job stress by working hours. As for the factors of job stress, the number of people working in a difficult physical environment exceeded 63% in the 53 h or more working group. The percentage of respondents who did not have autonomy was higher in the 41–68 h working group. Concerning job demand, we can interpret that the higher the score, the higher the job demand. In the group working more than 41 h, the score was more than 30 points, so the job demand was higher. The social support and adequate reward responses showed a U shape. Both had the lowest percentage of respondents who did not receive social support and adequate rewards in the 40 h working group. In other words, the ratio of social support and adequate reward was highest in the 40 h working group.

**Table 2:** Working hours organization and job stress N(%) or mean ± SD

	Working hours					
	Total	35–39	40	41–52	53–68	> 68
Total	23,197	644 (2.8)	10,497 (45.3)	7299 (31.5)	3653 (15.7)	1104 (4.8)
Difficult physical environment						
Yes	11,589 (50.0)	347 (53.9)	4473 (42.6)	3764 (51.6)	2307 (63.2)	698 (63.2)
No	11,608 (50.0)	297 (46.1)	6024 (57.4)	3535 (48.4)	1346 (36.8)	406 (36.8)
Job autonomy						
Yes	11,865 (51.1)	326 (50.6)	5640 (53.7)	3608 (49.4)	1732 (47.4)	559 (50.6)
No	11,332 (48.9)	318 (49.4)	4857 (46.3)	3691 (50.6)	1921 (52.6)	545 (49.4)
Job demand	29.3 ± 19.3	29.8 ± 19.2	27.1 ± 18.7	30.4 ± 19.3	32.7 ± 20.0	31.4 ± 19.7
Social support						
Yes	18,672 (80.5)	472 (73.3)	8944 (85.2)	5682 (77.8)	2777 (76.0)	797 (72.2)
No	4069 (17.5)	145 (22.5)	1404 (13.4)	1489 (20.4)	775 (21.2)	256 (23.2)
Non-applicable	456 (2.0)	27 (4.2)	149 (1.4)	128 (1.8)	101 (2.8)	51 (4.6)
Adequate reward						
Agree	8874 (38.3)	195 (30.3)	4645 (44.3)	2606 (35.7)	1141 (31.2)	287 (26.0)
Neither agree nor disagree	9309 (40.1)	270 (41.9)	3942 (37.6)	3059 (41.9)	1557 (42.6)	481 (43.6)
Disagree	5014 (21.6)	179 (27.8)	1910 (18.2)	1634 (22.4)	955 (26.1)	336 (30.4)

*Abbreviations:* standard deviation, SD

Tables 3 and 4 show the prevalence of depressive symptoms. The rate of depressive symptoms was 43.0% in the total study subjects, and the average of WHO’s well-being index in study subjects was 14.5.

**Table 3:** Prevalence of depressive symptoms by general characteristics

	No. of total subjects	No. of subjects with depressive symptoms	%
Total	23,197	9977	43.0
Gender			
Men	12,509	5349	42.8
Women	10,688	4628	43.3
Age			
19–29	3025	1160	38.3
30–39	6357	2447	38.5
40–49	7062	3049	43.2
50–59	4743	2233	47.1
60–69	1704	912	53.5
≥ 70	306	176	57.5
Education level			
Graduated high school or less	10,812	5387	49.8
College	11,833	4415	37.3
Higher than graduated college	552	175	31.7
Income (KRW)			
< 100	1227	652	53.1
100–199	8940	4255	47.6
200–299	7091	2894	40.8
≥ 300	5939	2176	36.6
Job category			
White collar	10,466	3739	35.7
Pink collar	5735	2576	44.9
Blue collar	6996	3662	52.3
Employment status			
Full-time employee	18,860	7735	41.0
Temporary employee	3229	1605	49.7
Day employee	1108	637	57.5
Physical problem			
Yes	10,836	5328	49.2
No	12,361	4649	37.6
Irregular working time			
Yes	3672	1798	49.0
No	19,525	8179	41.9

*Abbreviations:* Korean Won, KRW

The prevalence of depressive symptoms was the lowest in the 40 h, with the exception of the 35–39 h group, and the longer working time, the higher the prevalence, so that the U-shaped pattern presented. The prevalence of depressive symptoms was higher in the group working in an irregular working time and a difficult physical environment. The prevalence of depressive symptoms was high when job autonomy was low, there was no social support, and inadequate reward. In the depressed group, the score of job demand was 31.1 points higher than that of the non - depressed group.

Table 5 shows the prevalence ratios of depressive symptoms compared to 40 working hours per week as a reference. The prevalence of depressive symptoms was significantly higher than that of 40 h per week, 21% at 53–68 h and 14% at over 68 h after adjusted by gender, age, socioeconomic position, physical problems, and irregular working time. Model 3 was adjusted by including all of the job stress factors. The effect size was small, but statistically significant, that 16% at 53–68 h and 8% at over 68 h. In the effects of long working hours on depressive symptoms, job stress explained 20–40% when the working hours exceed 40 h per week. However, when the working hours were less than 39 h per week, job stress was hardly explained.

**Table 4:** Prevalence of depressive symptoms by working hours and job stress

	No. of total subjects	No. of subjects with depressive symptoms	%
Total	23,197	9977	43.0
Working hours			
35–39	644	311	48.3
40	10,497	3981	37.9
41–52	7299	3168	43.4
53–68	3653	1918	52.5
> 68	1104	599	54.3
Difficult physical environment			
Yes	11,589	5355	46.2
No	11,608	4622	39.8
Job autonomy			
Yes	11,865	4870	41.0
No	11,332	5107	45.1
Job demand (mean ± SD)	29.3 ± 19.3	31.1 ± 19.3	
Social support	19.3	19.3	
Yes	18,672	7422	39.7
No	4069	2320	57.0
Non-applicable	456	235	51.5
Adequate reward			
Agree	8874	2796	31.5
Neither agree nor disagree	9309	4338	46.6
Disagree	5014	2843	56.7

*Abbreviations:* standard deviation, SD

Table 6 shows the explanatory power of each of the job stress factors explaining depressive symptoms. Among them, social support explained 10–30% and accounted for the largest portion. Social support in the 35–39 h working group explained 18.8%, and in the 41–52 h working group explained 32.6%. Reward had the explanatory power of 15–30% in the 53 h or more working group. Depression was explained by 15.3% in the 53–68 h working group and 28.1% in the over 68 h working group. In the group that worked more than 68 h, the reward was the most important job stress factor by explaining almost 30%.

**Table 5:** The explanatory power of job stress explaining the association between working hours and depressive symptoms

	PR	(95% CI)	PR change (%)
Model 1			
35–39 h	1.25	(1.15–1.35)	
40 h	1		
41–52 h	1.13	(1.09–1.17)	
53–68 h	1.37	(1.33–1.42)	
> 68 h	1.33	(1.25–1.40)	
Model 2			
35–39 h	1.09	(1.01–1.18)	
40 h	1		
41–52 h	1.06	(1.03–1.10	
53–68 h	1.21	(1.16–1.25)	
> 68 h	1.14	(1.07–1.21)	
Model 3			
35–39 h	1.09	(1.01–1.18)	0.0
40 h	1		
41–52 h	1.04	(1.00–1.07)	41.0
53–68 h	1.16	(1.12–1.20)	23.6
> 68 h	1.08	(1.02–1.14)	40.9

Model 1: adjusted for age, gender

Model 2: adjusted for age, gender, education, income, physical problems, job category, employment status, working hours organization

Model 3: Model 2 + adjusted for job stress

PR change: percentage change in prevalence ratio = 100*((PR in baseline model (Model2)) – (PR in model adjusted for risk factor))/(PR in baseline model (Model2)) – 1)

*Abbreviations*: prevalence ratio, PR; confidence interval, CI

**Table 6:** The explanatory power of job stress factors explaining depressive symptoms

PR change (%)	Difficult physical environment	Job autonomy	Job demand	Social support	Reward
35–39 h	−0.9	3.2	−3.2	18.8	−8.5
40 h					
41–52 h	0.3	4.8	6.1	32.6	11.3
53–68 h	0.6	1.6	2.7	11.1	15.3
> 68 h	−1.1	1.8	−0.8	15.2	28.1

*Abbreviations*: prevalence ratio, PR

## Discussion

The percentage of depressive symptoms according to working hours showed a U shape, the lowest at 40 h. In the model adjusted for job stress and irregular working time, the 53–68 h group were 1.21 times higher and over 68 h working group were 1.14 times higher for depressive symptoms than the 40 h working group. In the pathway between long hours and depression, job stress explained 20–40%. Social support was 10–30%, which showed high explanatory power in all working hours. In the case of reward, the pathway was explained in the 53–68 and over 68 h working groups as 15.3 and 28.1%, respectively, which accounted for the largest portion.

We showed the long working hours could be an independent risk factor for depressive symptoms in employees. This was consistent with previous studies, but the effect size was smaller. According to a 5-year follow-up study of British civil servants, the odds ratio for major depressive episodes in people who worked more than 11 h per day was 2.43 (95% CI 1.11–5.30) times higher than those who worked 7–8 h per day [3]. In the same study subjects, depressive symptoms were 1.66 times (95% CI 1.06–2.61) and anxiety symptoms were 1.74 times (1.15–2.61) higher than those of people who worked more than 55 h a week compared to 35–40 h a week [4]. According to the longitudinal cohort surveyed over the 12 year period from 2001 to 2012, those working 49–59 h (− 0.52, 95% CI -0.74 to − 0.29, *p* < 0.001) and 60 h or more (− 0.47, 95% CI -0.77 to − 0.16, *p* = 0.003) had worse mental health than when they were working 35–40 h/week [12].

The effect size between work hours and depression in Korean studies varied from study to study but associations were observed. According to a study of 4662 full-time employees using K-NHANES IV (2007–2009), the depressive symptoms were 1.62 higher at ≥60 h per week than in those who worked < 52 h per week. (95% CI 1.20–2.18) [13]. In this study, depressive symptoms were measured by questioning whether they had depressive symptoms for more than 2 weeks last year. In addition, in a longitudinal study of 2733 full-time employees for 2010–2013, the odd of depressive symptoms (CES-D) were 1.57(95% CI 1.05 to 2.34) at > 68 h working group compared to 35–40 h [14]. In the study of 993 Korean manufacturing workers using WHO-5 tools, the odds ratio increased in possible depression group (≤ 28 points, OR 2.39 and 4.16) as working hours increased to 53–60, and 60 h, respectively [15].

The effect size of our study was smaller than other studies because of the different measurement tools, working hours category criteria and study subjects. We used WHO-5 to assess depressive symptoms. Compared with the DSM-IV used as the gold standard for major depression diagnosis, the WHO-5 cut-off score was restrictively equal to ≤28 (converted to 100 points) [16]. In our study, the cut-off score of the prevalence of depressive symptoms was 13 points (52 points converted to 100 points) as a screening tool for depression [17]. Although this could lower specificity, we used this criterion because our study tried to focus on psychosocial wellbeing, not strictly on major depression diagnosis. Relatively low specificity may cause non-differential misclassification and the informational bias, and the bias may dilute the prevalence ratio, which may have reduced the effect size.

Other studies have applied different criteria of working hours when exploring the association between working hours and health. In Korea, there have been studies classified according to the Korean Labor Standards Act (53–68 h, over 68 h) and the Industrial Accident Compensation Insurance Act for cardiovascular and cerebrovascular disease (over 60 h) [13, 15]. The criteria for defining long working hours in other countries were 11–12 h/day(3–4 h of overtime work) [3], greater than standard full-time hours(41–48, 49–59, over 60 h) [12], ≥40 h/week (8 h/day) [2], > 41 h/week [18] and ≥ 60 h/week [8]. The reference working hours used in these studies were 7–8 h/day [3, 4], 35–40 h/week [12, 18], 40 h/week [2], 35–47 h/week [15], < 52 h/week [13] and < 60 h/week [8], which varied from study to study.

Legally permissible working hours vary from country to country, and generally range between 35 and 40 h per week. Many countries are shortening their working hours. Germany is seeking working time reduction (Arbeitszeitverkuerzung) through labor and management agreements, and by using working time account (Arbeitszeitkonto), the employment is stabilized by increasing or decreasing working hours within a certain limit as demand increases or decreases. Thus, the average working time of 35–40 h per week is maintained. France [19] and Japan [20] are reducing working hours through the law. France legally sets working hours to 35 h per week and indirectly adjusts the working hours flexibly through labor-management agreements [21]. Unlike other countries, Korea does not set working hours through labor-management negotiations. Those who work less than 40 h in Korea are always considered to be part-time workers [22], so they will probably have fewer benefits than standard 40 h workers [23].

Based on these references, we categorized working hours into 35–39, 40, 41–52, 53–68, and > 68 h per week. The 40 h working group accounted for 45.3%, the largest proportion of the total subjects. However, considering Korea’s organizational culture, 40 h working time could be just a character in his/her employment contract, regardless of the actual working hours. This could cause differential misclassification and the information bias, which would have affected the effect size to be smaller.

Job stress is a risk factor of mental health including depression and anxiety [7]. In a study conducted using the Karasek’s job demand-control-support model, high job demands, low control, and low support were associated with depression and anxiety, respectively [24]. In the study of 240 workers at US public hospitals, social support at work was inversely related to depression, and the greater the social support, the lower the depressive symptoms (standardized coefficient 0.30) [25]. Social support was the most important factor in depression compared to other job stress factors, and our study also had high explanatory power in all working hours. The percentage of depressed symptom respondents was the lowest at 40 working hours (13.4%), and when working hours increased or decreased from the appropriate working hour, the percentage increased to finally showed the U shape. Those who worked 35–39 h were more in temporary and day employment than full-time employment. In a temporary or day employee, complex employment relationships may have resulted in job instability, lack of social support, and inadequate rewards. In Korean workplaces, regionalism and collectivistic work culture act as stress factors. Social support can be a buffer for job stress. However, there are also studies that the supervisors’ social support serves as a stress factor rather than a buffer [26].

In our study, the long working hours group had low satisfaction of reward and high depressive symptoms. The effort-reward imbalance model explains that there must be adequate compensation for effort. Given low reward despite high effort, the balance of the contract is broken and a strong stress response is triggered [27]. These stress responses can be larger in groups with long working hours. According to 12 prospective studies, depression increased about 1.8 times when exposed to high demand and low control or when subjects exerted high effort with low reward in the workplace [28]. According to the 17th labor and income panel study in Korea, the hourly wages of groups working 36–50 h and working more than 50 h per week were 14,000 KRW and 10,000 KRW, respectively. Wage per hour was lower in groups with long working hours [29]. People who work long hours in Korea have low wages per hour, so reward may be inadequate, which may cause depressive symptoms.

The strength of this study is to identify subareas of job stress that have a significant impact on depression symptoms. And we have identified by working hour groups which job stress factors explain much of depressive symptoms. Although there are other studies investigating the relationship between depressive symptoms and job stress and the relationship between depressive symptoms and working hours, few studies have investigated the three relationships at once. This study contains various confounding variables. Based on previous studies, we have adjusted for shift work, night work, income, employment status and physical problem which are found to be related to long working hours and negative emotional states. The study was not limited to a specific occupational group but was conducted as a large-scale sample representative of Korea.

The limitation of this study is that it is a cross-sectional study and therefore it is insufficient to prove causality. Reverse causality between long work hours and depressive symptoms could not be ruled out. Although cross-sectional studies can be used to estimate risk factors for depression, there is a limit to proving the temporal relationship. So it is necessary to conduct a cohort study or case-control study to clarify the causal relationship. Data were collected using self - administered questionnaires. Although a validated questionnaire on job stress and mental health was used, it is likely that it has been overestimated or underestimated because it is a subjective questionnaire. The WHO-5 tool used to assess depressive symptoms in this study can be used to screen for depression, but not for an accurate diagnosis of depression. In addition, workers working less than 39 h per week suffered more depressive symptoms than workers who worked 40 h. This may involve selection bias, so be careful in interpreting it.

## Conclusions

It is necessary to ensure adequate working hours for the mental health of workers. In the outline of long working hours affecting depressive symptoms, social support and inadequate reward were important factors affecting amount of job stress. Social support was the main explanatory factor of job stress related to working hours and depressive symptom. Therefore, we can infer that the social support of managers and colleagues in the workplace can play an important role in relieving depressive symptoms. It can help the mental health of workers through the managers’ leadership, respect of personalities, dispute resolution, organizational skills, and colleagues’ cooperation and support. In workers who work more than 68 h, reward accounted for a considerable portion of depressive symptom. When there was inadequate reward for hours worked, it was a risk factor for depressive symptoms. Therefore, for the mental health of employees, it is necessary to strengthen social support or give appropriate reward according to working hours, and ultimately to work in the suitable hours.

Long working hours can lead to a lack of recovery time, which can lead to depressive symptoms. Later, other factors such as physical and mental recovery need to be considered as the mechanisms of long working hours affecting depressive symptoms.

## Abbreviations

CI: Confidence Interval
EWCS: European Working Conditions Survey
ICD-10: International Statistical Classification 10th Revision
K-NHANES: Korea National Health and Nutrition Examination Survey
KWCS: Korea Working Conditions Survey
LFS: Labour Force Survey
OECD: Organization for Economic Cooperation and Development
OSHRI: Occupational Safety and Health Research Institute
PR: Prevalence ratio
WHO-5: WHO’s Well-Being Index
